# Current Status and Future Directions of mHealth Interventions for Health System Strengthening in India: Systematic Review

**DOI:** 10.2196/11440

**Published:** 2018-10-26

**Authors:** Abhinav Bassi, Oommen John, Devarsetty Praveen, Pallab K Maulik, Rajmohan Panda, Vivekanand Jha

**Affiliations:** 1 George Institute for Global Health, India New Delhi India; 2 University of New South Wales Sydney Australia; 3 George Institute for Global Health, India Hyderabad India; 4 George Institute for Global Health, Oxford University Oxford United Kingdom; 5 University of Oxford Oxford United Kingdom

**Keywords:** mHealth, telemedicine, health care system, India

## Abstract

**Background:**

With the exponential increase in mobile phone users in India, a large number of public health initiatives are leveraging information technology and mobile devices for health care delivery. Given the considerable financial and human resources being invested in these initiatives, it is important to ascertain their role in strengthening health care systems.

**Objective:**

We undertook this review to identify the published mobile health (mHealth) or telemedicine initiatives in India in terms of their current role in health systems strengthening. The review classifies these initiatives based on the disease areas, geographical distribution, and target users and assesses the quality of the available literature.

**Methods:**

A search of the literature was done to identify mHealth or telemedicine articles published between January 1997 and June 2017 from India. The electronic bibliographic databases and registries searched included MEDLINE, EMBASE, Joanna Briggs Institute Database, and Clinical Trial Registry of India. The World Health Organization health system building block framework was used to categorize the published initiatives as per their role in the health system. Quality assessment of the selected articles was done using the Cochrane risk of bias assessment and National Institutes of Health, US tools.

**Results:**

The combined search strategies yielded 2150 citations out of which 318 articles were included (primary research articles=125; reviews and system architectural, case studies, and opinion articles=193). A sharp increase was seen after 2012, driven primarily by noncommunicable disease–focused articles. Majority of the primary studies had their sites in the south Indian states, with no published articles from Jammu and Kashmir and north-eastern parts of India. Service delivery was the primary focus of 57.6% (72/125) of the selected articles. A majority of these articles had their focus on 1 (36.0%, 45/125) or 2 (45.6%, 57/125) domains of health system, most frequently service delivery and health workforce. Initiatives commonly used client education as a tool for improving the health system. More than 91.2% (114/125) of the studies, which lacked a sample size justification, had used convenience sampling. Methodological rigor of the selected trials (n=11) was assessed to be poor as majority of the studies had a high risk for bias in at least 2 categories.

**Conclusions:**

In conclusion, mHealth initiatives are being increasingly tested to improve health care delivery in India. Our review highlights the poor quality of the current evidence base and an urgent need for focused research aimed at generating high-quality evidence on the efficacy, user acceptability, and cost-effectiveness of mHealth interventions aimed toward health systems strengthening. A pragmatic approach would be to include an implementation research component into the existing and proposed digital health initiatives to support the generation of evidence for health systems strengthening on strategically important outcomes.

## Introduction

### Background

Progress in the management of communicable diseases and reproductive maternal and child health conditions, combined with demographic transition, have caused a shift in the burden of mortality and morbidity to noncommunicable diseases (NCDs) [1]. In India, the contribution of NCDs to deaths increased from 37.9% in 1990 to 61.8% in 2016 [2]. The pauci-symptomatic nature and the long-term management and medication availability requirements force a change in the approach to NCD care delivery from facility-based service to domiciliary care, in which the consumer is not in constant contact with the health care system. Integrated care delivery is required using a risk factor–based rather than a disease-specific approach, with the need for periodic reassessment and treatment modification. Rural areas are at particular disadvantage due to the inadequacy and maldistribution of workforce and services.

Technological innovations present the possibility of turning a mobile device into a key component of health care delivery. Reduction in cost of handsets and increase in network coverage has led to a rapid expansion of mobile phone ownership in India. The number of mobile connections in India has grown to over 1 billion with 42% of the subscribers living in rural areas [3]. Out of 650 million active mobile users in 2017, nearly 300 million had a smartphone [4].

Mobile health or mHealth, defined by the global observatory for eHealth as “medical and public health practice supported by mobile devices, such as mobile phones, patient monitoring devices, personal digital assistants, and other wireless devices” is increasingly being used to support NCD care delivery [5]. Potential advantages include reducing response time by using trained nonphysician health workers, providing decision support, minimizing variability in the quality of delivered care, and optimizing monitoring and patient engagement, eventually reducing the cost of care and improving outcomes [6].

A number of initiatives that use mobile devices for delivering health care are currently being developed and implemented in India [6-9]. Given the considerable financial and human resources being invested in planning, development, and implementation of these initiatives, it is critical to ascertain their role in strengthening health care systems.

### Objectives

We undertook this review to identify the published mHealth or telemedicine (provision of health care services using telecommunication technology) initiatives in India in the context of the health system building blocks and their potential for health systems strengthening. The review also presents the disease area, type of telecommunication device used, geographical distribution of the study sites, and target users of the innovation for these published mHealth initiatives in India. Finally, we highlight actions required for ensuring an effective role of mHealth interventions in strengthening the Indian health system.

## Methods

### Search Strategy

A search of the literature was done to identify mHealth or telemedicine articles published from India between January 1997 and June 2017. The electronic bibliographic databases and registries searched included MEDLINE, EMBASE, Joanna Briggs Institute EBP Database, World Health Organization’s (WHO) International Clinical Trials Registry Platform, ClinicalTrial.gov, IndMED, and Clinical Trial Registry of India. The key search terms used included device (smartphones, cell phone, mobile phone, tablet, personal digital assistant, laptop, personal computer); service (Interactive Voice Response, text message, global positioning system, videoconferencing); intervention (primary care, secondary care, tertiary care, disease prevention, disease control, disease management, risk factor control, telemedicine, and mHealth); diseases (NCDs, communicable diseases, maternal, and child health); and India. The list of subheadings (MeSH) and text-words used along with the detailed strategy used for searching the databases is provided as Multimedia Appendix 1. The information technology (IT) devices included computers, fixed-line phones, personal digital assistants, feature phones, smartphones, and wearables. Articles related to the health effects of IT devices were excluded from this review.

We included primary research articles (trials, quasi- experimental, pre- and postintervention, cohort studies, descriptive and analytical cross-sectional studies, exploratory studies, and protocols for trials or quasi-experimental studies); review articles (systematic and narrative reviews); system description studies (describing system architecture); case studies; and opinion papers. A database was developed using Microsoft Office Access interface, and information was abstracted in 2 stages.

### Stage 1

Year of publication and disease focus were abstracted for all the selected articles. The disease focus was classified under (1) communicable, maternal, perinatal, and nutritional conditions and (2) NCDs, using the WHO global health estimates classification [10].

### Stage 2

Research articles containing primary data were carried forward to the second stage. Data related to geographical location, devices used, intended target users, target health system domain, and type of mHealth app or tool used were extracted. Review articles, system description studies, case studies, and opinion papers that did not have specific information on the above-mentioned indicators were excluded at this stage.

The WHO health system building block framework was used to arrange the abstracted information under the following heads: (1) service delivery, (2) health workforce, (3) health information systems, (4) access to essential medicines, (5) financing, and (6) leadership or governance [11]. A framework developed by Labrique et al was used to classify the identified mHealth app or tool as per their types and uses [12]. We grouped all consumer-centric interventions by adding medical consultations offered through mobile technologies to the *client education and behavior change communication*. Health workers’ awareness and perception of mHealth were included under *provider training and education*.

The Cochrane risk of bias assessment tool was used to assess the risk of selection bias, reporting bias, performance bias, detection bias, and attrition bias in randomized controlled trials (RCTs). Agency for Healthcare Research and Quality standards score was used to arrive at a composite indicator of quality (good, fair, and poor) for RCTs. Study quality was considered (1) “good” if it met all criteria (low for each domain, as per Cochrane risk of bias tool); (2) “fair” if the risk of bias was high for 1 domain or unclear for 2 and unlikely to have biased the outcomes; and (3) “poor” if 2 or more criteria had high or unclear risk of bias likely to have affected the outcomes. The quality assessment for observational cohort, pre-post and cross-sectional studies was done using the National Institutes of Health, US Department of Health and Human Services quality assessment tool [13]. Two reviewers (AB and OJ) independently assessed the quality of the selected evidence. Any discordance in the selection, categorization, or quality assessment was resolved by discussion. The quality of the systematic reviews was assessed using the AMSTAR 2 checklist. AMSTAR 2 is a measurement tool created to assess the methodological quality of systematic reviews.

## Results

### Search Results

**Figure 1:**
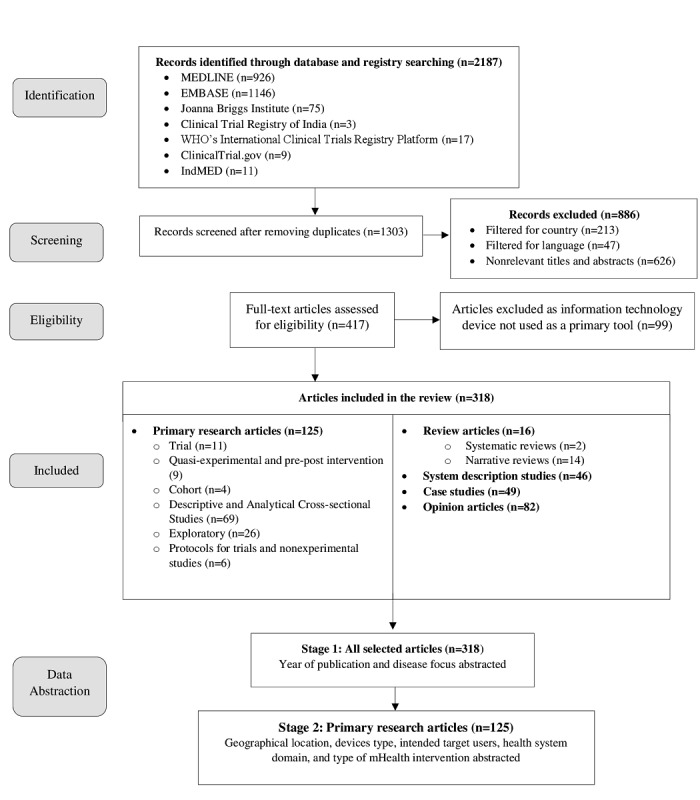
Preferred Reporting Items for Systematic Reviews and Meta-Analyses (PRISMA) flow diagram.

The combined search strategies yielded 2187 citations. After removing duplicates, a total of 1303 articles were screened for their relevance. Following the title and abstracts screening, a total of 886 articles were filtered out for criteria related to country, language, and nonrelevance. A total of 417 articles were selected for full-text evaluation. Exclusion of 99 articles not having mHealth or IT as the primary intervention resulted in the final selection of 318 articles (Figure 1).

**Figure 2:**
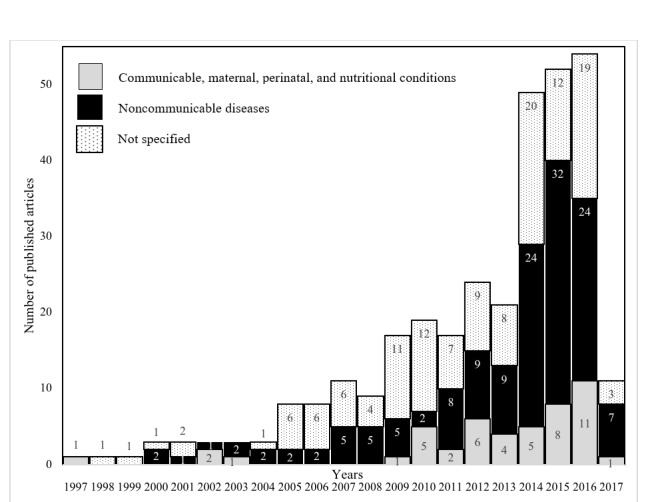
Year-wise distribution of the published articles and disease area.

Figure 2 shows the distribution of the articles and disease focus. Approximately 44.6% (142/318) of the selected articles had an NCD focus; 14.8% (47/318) were directed toward the domain of communicable, maternal, perinatal, and nutritional conditions; and the remaining 40.6% (129/318) addressed cross-cutting topics. The first 10 years (1997-2006) saw only a small number of articles with a focus on the role of telemedicine in improving the health services through medical consultations and communication between the health care providers. A sharp increase was seen after 2012, driven primarily by NCD-focused articles.

Table 1 presents distribution of the type of studies published between 1997 and 2017, divided into 5-year periods. Out of the 318 articles, more than 25.8% (82/318) were opinion-based articles, followed by 21.7% (69/318) descriptive and analytical cross-sectional studies. Less than 3.5% (11/318) followed an experimental design that allowed evaluation of the impact of interventions on the health outcomes. Most studies published between 1997 and 2006 were case studies or opinion articles.

**Figure 3:**
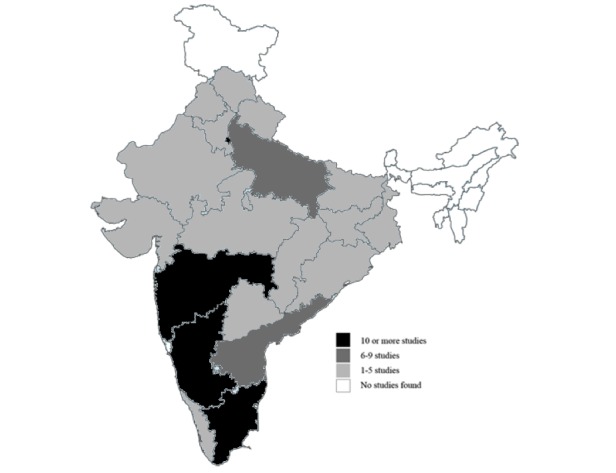
Geographical distribution of the study sites (n=125). Map source: Ministry of External Affairs, Government of India.

A majority of studies had been conducted in the south Indian states, with Tamil Nadu (27) and Karnataka (24) leading the list. Delhi (17) and Maharashtra (13) had the highest number of sites from the rest of the country. No articles were published from Jammu and Kashmir and north-east Indian states. Moreover, 7 published articles reported findings from multicentric studies. A map of India with the distribution of the study sites is available (Figure 3) [14].

**Figure 4:**
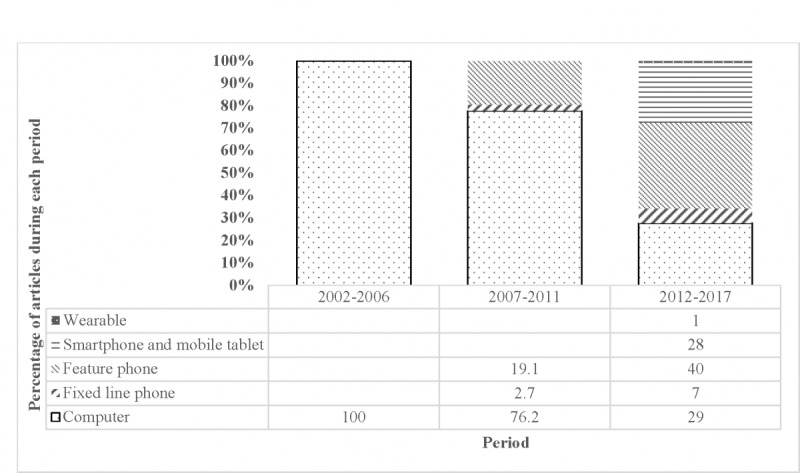
Changing preference of device used over time (n=125).

Figure 4 shows the devices used in the studies. Personal computers (desktops, notebooks) and fixed-line phones were the most commonly used tools until 2011. Articles using feature phones and smartphones as a technology device emerged after 2012.

A total of 125 articles provided information about the end users of the tool. Physicians were the most frequent end users 44.0% (55/125), followed by patients 26.4% (33/125) and general community 10.4% (13/125). Only 6.4% (8/125) of the initiatives aimed at engaging community health workers.

### Risk of Bias and Quality Assessment

Table 2 presents the risk of bias assessment for the selected RCTs. Overall, the methodological rigor of the included studies was poor. One study [15] had low risk of bias in all categories, whereas others had a high risk of bias in 2 or more categories. Moreover, 4 studies failed to provide enough information to allow assessment of risk of selection, performance, or detection bias (ie, unclear risk of bias).

Multimedia Appendices 2-4 provide details of quality assessment of the studies. More than 40% (44.8%, 56/125) of the cross-sectional studies had stated their research objectives or questions (Multimedia Appendix 2). Criterion related to description of the study population, demographics, clinical profile, and recruitment location was satisfied in 70% (48/69) of the studies. Sample size justification was not provided in more than 90% (62/69) of studies, with convenience sampling being the commonest approach. Only 15% (10/69) of the cross-sectional studies reported adjusting for potential confounders. Definition of the exposure (independent variables) and outcome measures (dependent variables) was present in approximately 60% (40/69) of the studies.

All the 4 cohort studies had stated their objectives and defined study populations (Multimedia Appendix 3). However, none of the studies had provided justification for the chosen sample size. All 4 studies used clearly defined, accepted methods for assessment of both exposure and control groups. Only 2 studies reported loss to follow-up.

**Table 1:** Year-wise distribution of the type of published articles.

Serial number	Study type	Period in years				Articles, n (%)
		1997-2001	2002-2006	2007-2011	2012-2017	
1	Trials	—^a^	—	2	9	11 (3.4)
2	Quasi-experimental and pre-post intervention	—	—	1	8	9 (2.8)
3	Cohort study	—	—	—	4	4 (1.3)
4	Descriptive and analytical cross-sectional studies	—	4	12	53	69 (21.7)
5	Exploratory	—	—	5	21	26 (8.2)
6	Protocols (trials and quasi-experimental)	—	—	1	5	6 (1.9)
7	Systematic and narrative reviews	—	—	3	13	16 (5)
8	System architectural	—	—	7	39	46 (14.5)
9	Case study	2	8	19	20	49 (15.4)
10	Opinion article	7	13	23	39	82 (25.8)

^a^Not applicable.

**Table 2:** Risk of bias assessment for randomized control trials.

Author	Random sequence generation	Allocation concealment	Blinding (participants and personnel)	Blinding (outcome assessment)	Selective reporting	Incomplete outcome data	Other sources of bias	AHRQ^a^ score
Arora et al, 2017 [15]	Low	Low	Low	Low	Low	Low	Low	Good
Jain et al, 2010 [16]	Low	Low	High	Low	Low	Low	Low	Fair
Sharma et al, 2011 [17]	High	High	High	Unclear	Low	Low	High^b^	Poor
Prasad et al, 2012 [18]	High	High	High	Unclear	Low	Low	Low	Poor
Ramachandran et al, 2013 [19]	Low	Low	High	Low	Low	Low	Low	Fair
Radhakrishnan et al, 2014 [20]	Low	Unclear	High	High	Unclear	Low	Low	Poor
Shet et al, 2014 [21]	Unclear	Low	Low	Low	Low	Low	Low	Fair
Kaur et al, 2015 [22]	Low	High	High	High	Low	Low	Low	Poor
Kumar et al, 2015 [23]	Low	High	High	Low	Low	Low	Low	Poor
Patnaik et al, 2015 [24]	High	High	High	High	High	High	High^c^	Poor
Limaye et al, 2017 [25]	Low	High	High	Low	Low	Low	Low	Poor

^a^AHRQ: Agency for Healthcare Research and Quality.

^b^Contamination and source of recruitment of the study subjects are not mentioned.

^c^High and unequal attrition rates noted between the 2 study arms.

**Table 3:** Classification of the mHealth initiatives based on different health systems’ building blocks and mHealth tools.

mHealth tools	World Health Organization health system building block classification					
	Service delivery	Health workforce	Medical products, vaccines, and technologies	Health information system	Leadership and governance	Total
Client education and behavior change communication	61	—^a^	—	1	1	63
Sensors and point-of-care diagnostics	2	—	11	—	—	13
Provider training and education	—	11	—	—	1	12
Provider-to-provider communication	5	5	—	—	—	10
Electronic decision support	3	5	1	—	—	9
Data collection and reporting	—	—	1	3	1	5
Registries or vital event tracking	1	—	—	4	—	5
Provider work planning and scheduling	—	4	—	—	—	4
Electronic health records	—	—	—	3	—	3
Supply chain management	—	—	1	—	—	1

^a^Not applicable.

Only 2 of the pre-post single group studies had a sufficient sample size, whereas others were feasibility studies with no sample size justification (Multimedia Appendix 4). Outcomes measures were defined in all, and 1 study had used a nonvalidated tool for outcomes measurement. Outcome assessor blinding was not reported in any of the pre-post studies. Quality assessment of the 2 systematic reviews revealed methodological flaws including a lack of risk of bias assessment (Multimedia Appendix 5 [26,27]).

### Health Systems Building Blocks

The classification in Table 3 is based on the primarily targeted health system building block and mHealth tool. Multimedia Appendix 6 provides details of the multiple health system building blocks targeted by each article. A majority of these articles had their focus on 1 (36.0%, 45/125) or 2 (45.6%, 57/125) blocks, most frequently service delivery and health workforce. Approximately 16.8% (21/125) of the articles targeted 3 building blocks, and only 2.4% (3/125) of the articles had initiatives that had bearings on 4 building blocks of the health system.

### Health Service Delivery

Nearly 57.6% (72/125) of the primary research articles had service delivery strengthening as a primary focus. Key activities covered included patient consultations, remote diagnosis, and follow-up through videoconferencing. Mobile phones were primarily used for treatment adherence reminders (text and Interactive Voice Response), appointment reminders, and behavior change messaging (n=8) [18,21,28-33]. Development process was described in 21 articles, 32 articles reported utility [16-25,30,31,34-57], and 20 articles reported user acceptability of the initiative [28,32,33,58-74]. Other aspects explored were technology-related perception of the end users (n=13) [29,75-85], patient satisfaction (n=2) [86,87], assessment of health care professional needs, and challenges related to health service delivery using IT (n=2) [88,89].

### Health Workforce

Nearly 20.0% (25/125) of the articles had health workforce as the primarily targeted health system domain. Establishment of a provider-to-provider communication through teleconsultations, remote trainings, and capacity building was the most common health workforce strengthening activity. Objectives included reporting changes in the knowledge scores of the health care workers following tele-education interventions (n=11) [90-100] or a survey of mHealth or telemedicine-related knowledge, attitude, and practice among the health care professionals without any intervention (n=8) [16,101-108]. Articled describing use of mobile apps for screening, referral, guideline-based care, and provider work planning appeared only after 2013. Development and utility of community health worker-centric interventions that facilitated task shifting for disease screening, referral, and health information dissemination were discussed in 5 articles [109-113].

### Essential Medical Products, Vaccines, and Technologies

Medical products and technology were the focus of 11.2% (14/125) of the selected articles, with nearly half relating to eye care [114-121], specifically using remotely operated technological tools for disease diagnosis (n=6) [115,118,122-125]. One study evaluated patient experience for health monitoring [126] and another evaluated a mobile-based vaccine management tool [127].

### Health Information

In total, 8.8% (11/125) of the articles evaluated strengthening of the health information system, with focus on vital event tracking, disease surveillance, and case notification in rural areas [128-137]. Articles were mostly related to communicable diseases, maternal, perinatal, and nutritional conditions. One study evaluated mobile- and tablet-based systems for collection of data related to behavior research [138].

### Leadership, Governance, and Financing

A total of 3 studies addressed leadership-, governance-, and financing-related issues. Finding the challenges related to the financing of the existing mHealth programs and the legal issues related to teleconsultations in India were the key objectives of these studies [139-141].

## Discussion

### Principal Findings

We describe, for the first time, the landscape of mHealth initiatives from a health system perspective from the second most populous country in the world that faces major challenges in health care delivery. The emerging evidence base around mHealth in India shows a progress from anecdotal telemedicine user stories to primary research articles, providing evidence on effectiveness in achieving the health objectives. The shift of focus of the mHealth initiatives over time toward NCDs is similar to the finding from China [142].

A notable finding was the concentration of mHealth solutions in a few states, with almost complete exclusion of the others, including some of the most underserved areas such as the north-eastern regions and Jammu and Kashmir, where mHealth might introduce great efficiency. A recent report by the Global Burden of Disease Study group pointed out at the heterogeneity of diseases and risk factors between Indian states [143]. Interstate variations in the structure and performance of health care delivery systems add to the challenge of last mile health care delivery. Therefore, it is important to test solutions in different states, especially the disadvantaged states that have the potential of experiencing the most transformative change.

The evolution in device choice may indicate changing consumer preferences in the contemporary mobile technology. However, the scientific basis for selecting these devices was not clearly articulated. Choice should take into account the technological know-how of end users, local health systems, nature of intervention, and availability of resources required to support the technology. The relevance of this knowledge becomes more important as these solutions are targeted to community health workers and patients to promote self-management and health promotion in communities.

Analysis in terms of the WHO health system building blocks revealed focus on service delivery and workforce strengthening, with relative neglect of health governance and health financing domains. Most of the reported mHealth interventions were being implemented as standalone solutions often with no health systems integration strategy. To reap maximal benefits, mHealth innovations should function as integrable tools that yield positive outcomes related to access, equity, quality, and responsiveness.

Client education, which increases access to health information, was the most widely used mHealth service delivery tool. However, contextual background to the health information that was being provided to the clients was not provided in the articles we reviewed. Similar findings in terms of the mHealth tools used emerged from China [142].

While assessing the studies for the methodological rigor, we found the use of nonvalidated instruments (survey and questionnaire) to be common. Sample size justification was provided only in a minority of reports. Use of convenience sampling has been a cause of prevalent skepticism related to mHealth interventions [144]. Another major flaw was the lack of a proper experimental design that allows generation of high-quality evidence. This combination of use of narrowly focused interventions in relatively small populations using loose experimental designs raises serious questions about internal as well as external validity of these studies, and this led to the use of a derisive term “pilotitis” to describe these mHealth studies.

### Limitations

Any review is only as good as the quality of the studies that are included. Studies published in biomedical literature only represent a subset of mHealth interventions as a fair number of studies are never submitted to academic journals. The mHealth apps that are available through the app stores were outside the scope of this review. Moreover, conducting a meta-analysis to provide estimates of clinical or cost-effectiveness was not possible due to the large differences in the methodologies used and outcome variables. Finally, while reporting the focus of the interventions, we used WHO’s health system building blocks and selected the primarily targeted health system domain. Caveat for interpreting these findings is that a number of interventions would have a synergistic effect on other health system domains. For instance, any intervention that is focused on capacity building of the health workforce would not only have an impact on the “health workforce” building block but would also improve the quality of care, thus strengthening the “service delivery.”

### Conclusions

In conclusion, mHealth initiatives are being increasingly tested to improve health care delivery in India. Despite the widespread perception that health care delivery capacity could be rapidly scaled up for achieving universal health coverage by leveraging the expanding mobile communications networks and high ownership of mobile devices, the quality of evidence remains suboptimal. Robust scientific evaluation of effectiveness through appropriately designed and sampled studies powered on clinical end points is critical for establishing the on-field appropriateness of mHealth initiatives. Our review highlights an urgent need for focused research aimed at generating high-quality evidence on efficacy and user acceptability of mHealth interventions aimed toward health systems strengthening considering contextual factors and size and specifics of the problems being addressed. We need well-designed, cost-effective studies to help policy makers use the finite health budgets to ensure maximum health benefits. A pragmatic approach would be to include an implementation research component into the existing and proposed digital health initiatives to support generation of evidence for health system strengthening on strategically important outcomes.

## Appendix

Multimedia Appendix 1 Search strategy for databases.

Multimedia Appendix 2 Quality assessment for cross-sectional studies.

Multimedia Appendix 3 Quality assessment for observational cohort.

Multimedia Appendix 4 Quality assessment for pre-post studies.

Multimedia Appendix 5 Quality assessment of systematic reviews (AMSTAR 2).

Multimedia Appendix 6 Study objectives, mHealth tool used, and health system framework classification of the selected articles.

## Abbreviations

IT: information technology
mHealth: mobile health
NCD: noncommunicable disease
RCT: randomized controlled trial
WHO: World Health Organization
