# Supplementary material for: Current Status and Future Directions of mHealth Interventions for Health System Strengthening in India: Systematic Review
Source: JMIR Mhealth Uhealth. 2018 Oct 26;6(10):e11440. doi: 10.2196/11440 (PMC6229512; doi:10.2196/11440)
Supplement: Multimedia Appendix 1 [file mhealth_v6i10e11440_app1.pdf]

Multimedia Appendix 1: Search Strategy for Databases

A) Search Strategy for MEDLINE through PubMed

Search	Query
**#1**	India[Tiab]
**#2**	(cell phones[Mh] or cellular telepho*[Tiab] or cell teleph*[Tiab] or cell phon*[Tiab] or mobile phon*[Tiab] or mobile teleph*[Tiab] or mobile devic*[Tiab] or iphone[Tiab] or ipad[Tiab] or android[Tiab])
**#3**	(blackberry[Tiab] or tablet pc[Tiab] or tablet[Tiab] or pager[Tiab] or personal digital assistant[Tiab] or pda[Tiab] or Electronic Mail[Mh] or email[Tiab] or e-mail[Tiab] or electronic mail[Tiab] or internet of thing*[Tiab])
**#4**	(telecommunications[Mh Terms] or telecommunication*[Tiab] or Mobile Applications[Mh] or mobile app*[Tiab] or smartphone app*[Tiab] or smart phone app*[Tiab] or tablet app[Tiab] or software[Tiab] or softwares)
**#5**	(Text Messaging[Mh] or Text messag*[Tiab] or texting[Tiab] or short messag*[Tiab] or short message service[Tiab] or SMS[Tiab] or multimedia messag*[Tiab] or multi media messag*[Tiab] or multi-media messag*[Tiab] or MMS[Tiab])
**#6**	(Interactive Voice Response[Tiab] or IVR[Tiab] or GPS[Tiab] or GPS track*[Tiab] or global positioning system*[Tiab] or GIS[Tiab] or geographic information system*[Tiab] or point of care devic*[Tiab] or Videoconferencing[Mh] or Videoconferencing [Tiab])))
**#7**	#2 OR #3 OR #4 OR #5 OR #6
**#8**	((Telemedicine[Mh] or mHealth[Tiab] or m-health[Tiab] or mobile health[Tiab] or mhealt*[Tiab] or mobile healthcare[Tiab] or mobile based health care[Tiab] or mobile health intervention[Tiab] or mhealth intervention[Tiab] or telemedicine*[Tiab] or tele-medicine[Tiab] or telehealth*[Tiab] or tele-health*[Tiab] or telecare[Tiab] or tele-care[Tiab] or telemonitoring[Tiab] or tele-monitoring[Tiab] or telemetry[Tiab] or tele-metry[Tiab] or eHealth[Tiab] or wireless telemedicine[Tiab])
**#9**	(mLearning[Tiab] or m-learning[Tiab] or mobile learning[Tiab] or mFinance[Tiab] or m-finance[Tiab] or mobile finance[Tiab])
**#10**	(Reminder Systems[Mh] or text reminder [Tiab] or text-reminder[Tiab] or reminder system[Tiab] or appointment reminder[Tiab])
**#11**	video consultation* [Tiab]
**#12**	(registries [Mh Terms] or medical regist*[Tiab] or electronic health records [Mh Terms] or electronic health record*[Tiab])
**#13**	(Chronic Disease[Mh] or Chronic illness*[Tiab] or NCD[Tiab] or NCDs or non-communicable diseas*[Tiab] or non communicable diseas*[Tiab] or diabetes[Tiab] or cancer[Tiab] or cardiovascular diseas*[Tiab] or respiratory diseas*[Tiab]

	or Mental disorders[Mh] or mental disorder[Tiab] or tobacco[Tiab] or alcohol[Tiab] or physical activity[Tiab] or unhealthy diet[Tiab])
**#14**	(Communicable Diseases[Mh] or Communicable diseas*[Tiab] or Infectious diseas*[Tiab] or Infection[Tiab] or Infections[Tiab] or tb[Tiab] or tuberculosis[Tiab] or AIDS[Tiab] or HIV[Tiab] or Malaria[Tiab] or STD[Tiab] or sexually transmitted diseas*[Tiab])
**#15**	(MNCH[Tiab] or maternal health[Tiab] or child health[Tiab] or neonatal health[Tiab] or maternal health[Tiab] or child health[Tiab] or adolescent health[Tiab])
**#16**	(Life Style[Mh] or Risk Reduction Behavior[Mh] or Risk Factors[Mh] or risk factor*[Tiab] or vector borne[Tiab] or vector-borne[Tiab])
**#17**	(Primary prevention[Mh] or Primary prevention[Tiab] or Primary Intervention[Tiab] or Primary health*[Tiab] or disease prevention[Tiab] or communicable disease prevention[Tiab] or infection prevention[Tiab] or infectious disease prevention[Tiab] or chronic illness prevention[Tiab] or NCD prevention[Tiab] or NCDs prevention[Tiab] or non communicable disease prevention[Tiab] or disaster prevention[Tiab] or injury prevention[Tiab])
**#18**	(Secondary prevention[Mh] or Secondary prevention[Tiab])
**#19**	(vaccine[Tiab] or vaccination[Tiab])
**#20**	(Communicable Disease Control[Mh] or disease control*[Tiab] or communicable disease control[Tiab] or infection control[Tiab] or chronic illness control[Tiab] or NCDs control[Tiab] or NCD control[Tiab] or non communicable disease control[Tiab] or vector control[Tiab] or risk-factor control[Tiab] or risk-factor control[Tiab])
**#21**	(Disease Management[Mh] or disease management*[Tiab] or communicable disease management[Tiab] or chronic illness management[Tiab] or NCDs management[Tiab] or NCD management[Tiab] or non communicable disease management[Tiab] or risk factor management[Tiab] or risk-factor management[Tiab] or risk-factors management[Tiab] or lifestyle management[Tiab] or life-style management[Tiab] or disaster management[Tiab] or injury prevention[Tiab] or primary health care management[Tiab] or primary healthcare management[Tiab])
**#22**	(Health Care[Tiab] or point of care systems[Mh Terms] or healthcare deliver*[Tiab] or delivery of healthcare[Tiab] or health care[Tiab] or healthcare[Tiab] or health care system*[Tiab] or healthcare system*[Tiab])
**#23**	(Leadership[Mh] or Healthcare Financing[Mh] or Health Manpower[Mh] or Health information systems[Mh] or Health Services Research[Mh] or Delivery of Health Care[Mh] or Outcome Assessment Health Care[Mh] or Health Policy*[Mh])

**#24**	(Governance[Tiab] or mGovernance[Tiab] or m-governance[Tiab] or mobile governance[Tiab] or mobile health laws[Tiab] or mobile health regulations[Tiab] or remote monitoring[Tiab] or patient monitoring[Tiab] or patient management[Tiab] or public health surveillance[Tiab] or monitoring of software[Tiab] or registration of software[Tiab] or Supply-chain management[Tiab] or supply chain management[Tiab] or tracking system[Tiab] or impact assessment[Tiab] or mobile health evaluation[Tiab] or cost-effectiveness[Tiab] or health financing[Tiab] or task shifting[Tiab] or human resource[Tiab] or health workforce[Tiab] or task-shifting[Tiab] or health information service*[Tiab] or health information sharing[Tiab] or information management[Tiab] or health information management[Tiab] or record maintenance[Tiab] or logistic management[Tiab] or Health technology[Tiab] or health care technology[Tiab] or healthcare technology[Tiab])
**#25**	(clinical decision-making[Tiab] or clinical decision making[Tiab] or clinical decision support system*[Tiab] or CDSS[Tiab] or treatment support[Tiab] or diagnostic support[Tiab] or emergency medical response[Tiab])
**#26**	(Counselling[Mh] or Health Literacy[Mh] or Patient Compliance[Mh] or Patient Education as Topic[Mh] or Risk Reduction Behavior[Mh] or Self-care[Mh] or Health promotion[Tiab] or adherence[Tiab] or health communication[Tiab] or patient awareness[Tiab] or patient education[Tiab] or personal health record[Tiab] or self-management[Tiab]))
**#27**	#8 OR #9 OR #10 OR #11 OR #12 OR #13 OR #14 OR #15 OR #16 OR #17 OR #18 OR #19 OR #20 OR #21 OR #22 OR #23 OR #24 OR #25 OR #26 OR
**#28**	#27 AND #7 AND #1

B) Search Strategy for EMBASE

**Query**
India:ab,ti AND (‘telehealth’/exp OR ‘telemedicine’ OR ‘cell phones’ OR ‘mobile phone’ OR ‘telemedicine’ OR ‘mHealth’ OR ‘mobile healthcare’)

C) Search Strategy for JBI Database of Systematic Reviews and Implementation Reports

**Query**
India ; cell phones or cellular telepho* or cell teleph* or cell phon* or mobile phon* or mobile teleph* or mobile devic* or iphone or ipad or android or telemedicine or mHealth or m-health or mobile health or mhealt* or mobile healthcare or mobile based health care or mobile health intervention or mhealth intervention or telemedicine* or tele-medicine or telehealth* or tele-health* or telecare or tele-care or telemonitoring or tele-monitoring or telemetry or tele-metry or eHealth or wireless

telemedicine; Chronic Disease or Chronic illness* or NCD or NCDs or non-communicable diseas* or non communicable diseas* or diabetes or cancer or cardiovascular diseas* or respiratory diseas* or Mental disorders or mental disorder or tobacco or alcohol or physical activity or unhealthy diet or Communicable Diseases or Communicable diseas* or Infectious diseas* or Infection or Infections or tb or tuberculosis or AIDS or HIV or Malaria or STD or sexually transmitted diseas* or primary prevention or primary prevention or primary health* or disease prevention or communicable disease prevention or infection prevention or infectious disease prevention or chronic illness prevention or NCD prevention or NCDs prevention or non communicable disease prevention or disaster prevention or injury prevention or disease management or disease management* or communicable disease management or chronic illness management or NCDs management or NCD management or non communicable disease management or risk factor management or risk-factor management or risk-factors management or lifestyle management or life-style management or disaster management or injury prevention or primary health care management or primary healthcare management

D) Search Strategy for JBI Database of Systematic Reviews and Implementation Reports

Query
Type of Trial=Observational OR Interventional| Study Design=Single Arm Trial OR Non-randomized, Placebo Controlled Trial OR Non-randomized, Active Controlled Trial OR Non-randomized, Multiple Arm Trial OR Randomized, Parallel Group Trial OR Randomized, Parallel Group, Placebo Controlled Trial OR Randomized, Parallel Group, Active Controlled Trial OR Randomized, Parallel Group, Multiple Arm Trial OR Cluster Randomized Trial | Phase of Trial=N/A| Primary Sponsor of Trial=ALL| Recruitment Status of Trial(TOTAL)=ALL| State where study is conducted=ALL| Keyword=MOBILE HEALTH

E) Search Terms used for WHO's International Clinical Trials Registry Platform, ClinicalTrials.gov and IndMED

Keywords
Mobile health; mHealth; eHealth; Telemedicine
