# Supplementary material for: Current Status and Future Directions of mHealth Interventions for Health System Strengthening in India: Systematic Review
Source: JMIR Mhealth Uhealth. 2018 Oct 26;6(10):e11440. doi: 10.2196/11440 (PMC6229512; doi:10.2196/11440)
Supplement: Multimedia Appendix 2 [file mhealth_v6i10e11440_app2.pdf]

Multimedia Appendix 2: Quality Assessment for Cross-Sectional Studies

Author	Objective clearly stated	Study population specified	Recruitment from the same population	Sample size justification	Independent variable clearly defined	Dependent variable clearly defined	Potential confounders measured or adjusted
Verma et al., 2009 [28]	No	No	No	No	No	Yes	No
Manoharan et al., 2012 [29]	No	No	No	No	No	Yes	No
Sidney et al., 2012 [31]	Yes	Yes	Yes	No	Yes	Yes	No
Kaliyadan et al., 2009 [34]	Yes	No	No	No	Yes	Yes	No
Singh et al., 2010 [36]	No	Yes	Yes	No	No	No	No
Singh et al., 2010 [37]	No	Yes	No	No	Yes	No	No
Alexander et al., 2011 [38]	Yes	Yes	No	No	Yes	Yes	No
Agrawal et al., 2012 [39]	No	Yes	Yes	No	Yes	No	No
Rachapelle et al., 2013 [43]	Yes	Yes	No	No	Yes	Yes	Yes
Agrawal et al., 2014 [44]	No	No	No	No	Yes	Yes	No
Gupta et al., 2016 [51]	Yes	Yes	Yes	No	No	Yes	No
Robin et al., 2016 [57]	Yes	Yes	Yes	No	No	Yes	No
Bali et al., 2007 [58]	No	No	No	No	No	No	No
Mohan et al., 2012 [60]	Yes	Yes	Yes	No	Yes	Yes	No
Elangovan et al., 2013 [61]	Yes	Yes	Yes	No	Yes	Yes	No
Ramkumar et al., 2013 [62]	Yes	Yes	Yes	No	Yes	Yes	No
Balasinorwala et al., 2014 [63]	Yes	No	No	No	No	Yes	No
Chandra et al., 2014 [64]	Yes	Yes	Yes	No	Yes	Yes	No
DeSouza et al., 2014 [66]	Yes	Yes	Yes	No	Yes	Yes	Yes
Gupta et al., 2014 [67]	Yes	No	No	No	No	Yes	Yes
Agarwal et al., 2015 [68]	Yes	Yes	No	No	No	Yes	No
Priscilla et al., 2015 [69]	Yes	No	No	No	Yes	Yes	No
Sureshkumar et al., 2015 [70]	Yes	Yes	Yes	No	Yes	No	No
Monica et al., 2017 [73]	Yes	Yes	Yes	No	No	Yes	No

Author	Objective clearly stated	Study population specified	Recruitment from the same population	Sample size justification	Independent variable clearly defined	Dependent variable clearly defined	Potential confounders measured or adjusted
Meher et al., 2009 [75]	Yes	Yes	No	No	Yes	No	No
Shet et al., 2010 [76]	Yes	Yes	Yes	No	Yes	No	No
Laxmi et al., 2014 [77]	Yes	Yes	Yes	No	Yes	Yes	No
Priyaa et al., 2014 [78]	Yes	Yes	Yes	No	Yes	Yes	No
Jain et al., 2015 [79]	Yes	Yes	Yes	No	Yess	No	No
Ramachandran et al., 2015 [80]	Yes	Yes	Yes	No	No	Yes	No
Acharya et al., 2016 [82]	Yes	No	No	No	Yes	No	No
Mudgapalli et al., 2016 [83]	Yes	Yes	Yes	No	Yes	Yes	No
Parthaje et al., 2016 [84]	Yes	Yes	Yes	Yes	Yes	Yes	No
Kumari et al., 2006 [86]	Yes	Yes	Yes	No	Yes	No	No
Paul et al., 2006 [87]	Yes	Yes	Yes	No	Yes	No	No
Deodhar et al., 2002 [90]	No	Yes	Yes	No	No	Yes	No
Jialdasani et al., 2006 [91]	Yes	No	No	No	No	Yes	No
Mahadevan et al., 2012 [93]	No	Yes	Yes	No	No	Yes	No
Agrawal et al., 2014 [94]	Yes	Yes	Yes	No	Yes	No	No
Chanani et al., 2016 [98]	Yes	Yes	Yes	No	Yes	Yes	No
Dorwal et al., 2016 [99]	Yes	Yes	Yes	No	Yes	Yes	No
Kaliyadan et al., 2016 [100]	Yes	Yes	Yes	No	Yes	Yes	No
George et al., 2007 [101]	Yes	Yes	Yes	No	No	No	No
Agarwal et al., 2010 [102]	No	Yes	Yes	No	No	No	No
Gour et al., 2010 [104]	Yes	Yes	Yes	No	Yes	No	No
Boringi et al., 2015 [105]	Yes	Yes	Yes	No	Yes	Yes	No
Ganapathy et al., 2016 [106]	Yes	No	No	No	Yes	Yes	Yes
Patil et al., 2016 [107]	Yes	Yes	Yes	No	Yes	Yes	No
Vivek et al., 2016 [108]	No	Yes	Yes	No	No	No	No
Perumalsamy et al., 2007 [114]	Yes	Yes	No	No	Yes	No	No
Srinivasan et al., 2012 [115]	Yes	Yes	Yes	No	Yes	Yes	Yes

Author	Objective clearly stated	Study population specified	Recruitment from the same population	Sample size justification	Independent variable clearly defined	Dependent variable clearly defined	Potential confounders measured or adjusted
Gupta et al., 2013 [116]	Yes	No	No	Yes	Yes	Yes	Yes
Raman et al., 2014 [117]	Yes	No	No	No	No	Yes	No
Sreelatha et al., 2014 [118]	Yes	No	No	No	No	No	No
Rajalakshmi et al., 2015 [119]	Yes	Yes	Yes	Yes	Yes	Yes	Yes
Ryan et al., 2015 [120]	Yes	Yes	No	Yes	Yes	Yes	Yes
Imtiaz et al., 2017 [121]	Yes	Yes	Yes	Yes	Yes	Yes	No
Bedekar et al., 2014 [122]	Yes	Yes	Yes	No	No	Yes	Yes
Malhotra et al., 2014 [123]	Yes	No	No	No	Yes	Yes	Yes
Singh et al., 2014 [124]	Yes	Yes	Yes	No	No	Yes	No
Patterson et al., 2015 [125]	Yes	No	No	No	Yes	Yes	No
Shrivastav et al., 2014 [126]	Yes	No	No	No	No	Yes	No
Giduthuri et al., 2014 [129]	Yes	Yes	Yes	No	Yes	Yes	No
Radhakrishna et al., 2014 [130]	Yes	Yes	No	No	No	Yes	No
Velayutham et al., 2015 [133]	Yed	Yes	Yes	No	No	No	No
Kazi et al., 2016 [134]	Yes	No	No	No	No	Yes	No
Modi et al., 2016 [135]	Yes	Yes	Yes	Yes	Yes	Yes	No
Gupta et al., 2017 [137]	Yes	Yes	Yes	No	Yes	Yes	Yes
Meher et al., 2013 [140]	Yes	Yes	Yes	No	No	Yes	No
Criteria 3 (participation rate of eligible person), 6 (measurement of exposure, prior to the outcome), 7 (sufficiency of timeframe to see an association between exposure and outcome), 8 (categories of exposure), 10 (frequency of assessment of exposure), 12 (blinding of outcomes assessors) and 13 (lost to follow-up after baseline) were not relevant to the methodology of the cross-sectional studies, and hence not used.							
