# Supplementary material for: Current Status and Future Directions of mHealth Interventions for Health System Strengthening in India: Systematic Review
Source: JMIR Mhealth Uhealth. 2018 Oct 26;6(10):e11440. doi: 10.2196/11440 (PMC6229512; doi:10.2196/11440)
Supplement: Multimedia Appendix 4 [file mhealth_v6i10e11440_app4.pdf]

Multimedia Appendix 4: Quality Assessment for Pre-Post studies

**Criteria**	Madan et al., 2016 [53]	Ajay et al., 2016 [71]	Thukral et al., 2014 [95]	Khanna et al., 2015 [96]	Maulik et al., 2017 [113]
1. Was the study question or objective clearly stated?	Yes	Yes	Yes	No	Yes
2. Were eligibility/selection criteria for the study population pre-specified and clearly described?	No	Yes	No	Yes	Yes
3. Were the participants in the study representative of those who would be eligible for the test/service/intervention in the general or clinical population of interest?	No	Yes	Yes	Yes	Yes
4. Were all eligible participants that met the pre-specified entry criteria enrolled?	No	Yes	CD	Yes	Yes
5. Was the sample size sufficiently large to provide confidence in the findings?	No	Yes	No	No	Yes
6. Was the test/service/intervention clearly described and delivered consistently across the study population?	No	Yes	Yes	Yes	Yes
7. Were the outcome measures pre-specified, clearly defined, valid, reliable, and assessed consistently across all study participants?	Yes	Yes	Yes	No	Yes
8. Were the people assessing the outcomes blinded to the participants' exposures/interventions?	No	No	No	No	No
9. Was the loss to follow-up after baseline 20% or less? Were those lost to follow-up accounted for in the analysis?	Yes	Yes	Yes	Yes	Yes
10. Did the statistical methods examine changes in outcome measures from before to after the intervention? Were statistical tests done that provided p values for the pre-to-post changes?	Yes	Yes	Yes	Yes	Yes
11. Were outcome measures of interest taken multiple times before the intervention and multiple times after the intervention (i.e., did they use an interrupted time-series design)?	No	Yes	No	No	Yes
12. If the intervention was conducted at a group level (e.g., a whole hospital, a community, etc.) did the statistical analysis take into account the use of individual-level data to determine effects at the group level?	Yes	Yes	Yes	No	Yes
