# Supplementary material for: Current Status and Future Directions of mHealth Interventions for Health System Strengthening in India: Systematic Review
Source: JMIR Mhealth Uhealth. 2018 Oct 26;6(10):e11440. doi: 10.2196/11440 (PMC6229512; doi:10.2196/11440)
Supplement: Multimedia Appendix 6 [file mhealth_v6i10e11440_app6.pdf]

Multimedia Appendix 6: Study Objectives, mHealth Tool Used and Health System Framework Classification of the Selected Articles

#Details of Health System Building Blocks: 1-Service delivery; 2-Health workforce; 3-Health information system; 4-Medical products, vaccines, and technologies; 5-Health financing system; 6-Leadership and governance

**Author**	**Study Objective**	**mHealth Tools**	**Health System Building Blocks#**						**Primary Health System Building Block**
			**1**	**2**	**3**	**4**	**5**	**6**	
Arora et al., 2017 [15]	To determine the effectiveness of telephone-based management of pressure ulcers in people with spinal cord injury	Client education and behaviour change communication	*	*					Service delivery
Jain et al., 2010 [16]	To compare gains in knowledge and skills of neonatal resuscitation using tele-education instruction versus conventional classroom teaching	Provider training and education		*					Health workforce
Sharma et al., 2011 [17]	To compare the effectiveness of short messaging service (SMS) and pamphlets in imparting health education to mothers of preschool children	Client education and behaviour change communication	*						Service delivery
Prasad et al., 2012 [18]	To evaluate the effect of appointment reminders, sent as SMS to patients attending outpatient clinics at a dental research center	Client education and behaviour change communication	*						Service delivery
Ramachandran et al., 2013 [19]	To assess whether mobile phone messaging that encourages lifestyle change could reduce incident type 2 diabetes mellitus (DM) in Indian men with impaired glucose tolerance	Client education and behaviour change communication	*						Service delivery
Radhakrishnan et al., 2014 [20]	To assess the effectiveness of a home-based exercise programme with IT support in people with metabolic syndrome	Client education and behaviour change communication	*						Service delivery
Shet et al., 2014 [21]	To assess whether customized mobile phone reminders would improve adherence to antiretroviral therapy (ART) and decrease virological failure among human immunodeficiency virus (HIV), infected patients	Client education and behaviour change communication	*						Service delivery
Kaur et al., 2015 [22]	To evaluate the impact of introducing telephonic consultation and follow-up in patients with DM	Client education and behaviour change communication	*	*					Service delivery

**Author**	**Study Objective**	**mHealth Tools**	**Health System Building Blocks#**						**Primary Health System Building Block**
			**1**	**2**	**3**	**4**	**5**	**6**	
Kumar et al., 2015 [23]	To assess the effect of mobile phone reminders on screening yield during opportunistic screening for type 2 DM in primary health care setting	Client education and behaviour change communication	*						Service delivery
Patnaik et al., 2015 [24]	To develop and evaluate an intervention model for the reduction of stress among patients with DM	Client education and behaviour change communication	*	*					Service delivery
Limaye et al., 2017 [25]	To investigate a virtual assistance based lifestyle intervention to reduce risk factors for type 2 DM in young employees from IT industry	Client education and behaviour change communication	*	*					Service delivery
Verma et al., 2009 [28]	To assess the feasibility of making a diagnosis of adnexal and orbital diseases through tele-ophthalmology	Provider-to-provider communication	*	*					Service delivery
Manoharan et al., 2012 [29]	To demonstrate the frequency of calls and the nature of questions raised by HIV patients over a telephonic helpline for improving ART adherence	Client education and behaviour change communication	*	*					Service delivery
Rodrigues et al., 2012 [30]	To assess the influence of mobile phone reminders on ART adherence and participants' experiences with the intervention	Client education and behaviour change communication	*						Service delivery
Sidney et al., 2012 [31]	To assess the perceived usefulness and acceptability of mobile phone reminders to support adherence to ART treatment	Client education and behaviour change communication	*						Service delivery
Swendeman et al., 2015 [32]	Development and pilot testing of daily interactive voice response (IVR) calls to support ART adherence	Client education and behaviour change communication	*						Service delivery
Thakkar et al., 2016 [33]	To develop and customize a SMS program supporting behaviour and treatment adherence in coronary heart disease	Client education and behaviour change communication	*	*					Service delivery
Kaliyadan et al., 2009 [34]	To evaluate the clinical profiles of cases referred for tele-dermatology, and to compare different modalities of tele-dermatology consultations	Client education and behaviour change communication	*	*					Service delivery
De Costa et al., 2010 [35]	To presents the study protocol for a trial, aiming to evaluate the influence of mobile phone reminders on ART adherence	Client education and behaviour change communication	*						Service delivery

Author	Study Objective	mHealth Tools	Health System Building Blocks#						Primary Health System Building Block
			1	2	3	4	5	6	
Singh et al., 2010 [36]	To show utility of telemedicine for providing health services to children	Provider-to-provider communication	*	*					Service delivery
Singh et al., 2010 [37]	To assess the application of telemedicine services for diagnosis and management of paediatric illnesses, through prospective analyses of electronic databases	Provider-to-provider communication	*	*					Service delivery
Alexander et al., 2011 [38]	A descriptive study on the users and utility of HIV helpline	Client education and behaviour change communication	*						Service delivery
Agrawal et al., 2012 [39]	To assess the implementation and socioeconomic impact of a call centre in providing healthcare delivery for patients with head and spinal injuries	Client education and behaviour change communication	*	*					Service delivery
Kesavadev et al., 2012 [40]	To assess the effectiveness, safety, and costs of a tele-management system based health care for type 2 DM patients	Client education and behaviour change communication	*						Service delivery
Schneider et al., 2012 [41]	To evaluate retention using telephone technology to contact participants for follow-up surveys, in comparison to using in-person interviews at their workplace	Client education and behaviour change communication	*						Service delivery
Praveen et al., 2013 [42]	To presents the study protocol for a multifaceted strategy using mobile technology to assist rural primary healthcare doctors and frontline health workers in cardiovascular disease (CVD) risk management	Electronic decision support	*	*		*			Service delivery
Rachapelle et al., 2013 [43]	To assess the cost-effectiveness of a telemedicine diabetic retinopathy screening program	Electronic decision support	*	*					Service delivery
Agrawal et al., 2014 [44]	To understand the feasibility and effectiveness of tele-recruitment of blood donors	Client education and behaviour change communication	*						Service delivery
Arora et al., 2015 [45]	To presents the study protocol for assessing the effectiveness and cost-effectiveness of telephone based support versus usual care for treatment of pressure ulcers in people with spinal cord injuries	Client education and behaviour change communication	*	*					Service delivery
Smith et al., 2015 [46]	To assess the potential for using mHealth in CVD by exploring experiences and challenges of current management, current	Client education and behaviour change communication	*	*					Service delivery

Author	Study Objective	mHealth Tools	Health System Building Blocks[#]						Primary Health System Building Block
			1	2	3	4	5	6	
	mobile phone use and expectations of and barriers to mobile phone use								
Sureshkumar et al., 2015 [47]	To present the protocol for development and evaluation of a smartphone enabled, caregiver supported educational intervention for management of physical disabilities following stroke	Client education and behaviour change communication	*	*					Service delivery
Thomas et al., 2015 [48]	To develop an open pilot trial for an HIV prevention intervention integrating mobile phone technology for male sex workers	Client education and behaviour change communication	*						Service delivery
Balakrishnan et al., 2016 [49]	To assess the effectiveness of the continuum of care services mHealth platform for strengthening the delivery of maternal, newborn and child health (MNCH) services	Registries/vital event tracking	*	*		*			Service delivery
Ganesan et al., 2016 [50]	To determine the effect of Stepathlon, an international, mHealth intervention, on physical activity, sitting, and weight	Client education and behaviour change communication	*						Service delivery
Gupta et al., 2016 [51]	To assess the utility of web based mobile technology monitoring tool, for ensuring linkages, and tracking of HIV exposed child	Client education and behaviour change communication	*	*	*				Service delivery
Jadhav et al., 2016 [52]	To assess the effectiveness of reinforcement of oral health education through SMS in mobile phones	Client education and behaviour change communication	*						Service delivery
Madan et al., 2016 [53]	To monitor change in tooth brushing behaviour after an intervention using smartphone video camera	Client education and behaviour change communication	*						Service delivery
Pfammatter et al., 2016 [54]	To examine whether mHealth DM intervention improved fruit, vegetable, and fat intakes and exercise	Client education and behaviour change communication	*						Service delivery
Prinja et al., 2016 [55]	To present the protocol for impact assessment and cost-effectiveness of mHealth application used by CHWs for MNCH care services	Client education and behaviour change communication	*						Service delivery
Reynolds et al., 2016 [56]	To present a protocol for evaluating a nurse delivered mHealth intervention for women with HIV and psychosocial risk factors	Client education and behaviour change communication	*	*					Service delivery

**Author**	**Study Objective**	**mHealth Tools**	**Health System Building Blocks[#]**						**Primary Health System Building Block**
			**1**	**2**	**3**	**4**	**5**	**6**	
Robin et al., 2016 [57]	To evaluate the effectiveness of tele-consultation by nurses using an integrated call centre	Client education and behaviour change communication	*	*					Service delivery
Bali et al., 2007 [58]	To ascertain the acceptability and feasibility of consultation by mobile phone in a rural area	Client education and behaviour change communication	*	*					Service delivery
Mishra et al., 2009 [59]	To assess the feasibility of telephonic follow-up in post-operative cases of thyroid and parathyroid diseases	Client education and behaviour change communication	*	*					Service delivery
Mohan et al., 2012 [60]	To implement comprehensive DM screening, prevention, and treatment using a combination of telemedicine and personalized care in rural areas	Client education and behaviour change communication	*	*					Service delivery
Elangovan et al., 2013 [61]	To analyze the mobile phone usage and its effectiveness in tuberculosis directly observed treatment short course treatment	Client education and behaviour change communication	*	*					Service delivery
Ramkumar et al., 2013 [62]	To assess the feasibility of conducting auditory brainstem response testing in a mobile van with satellite connectivity, with the help of trained health workers	Sensors and point-of-care diagnostics	*	*					Service delivery
Balasinorwala et al., 2014 [63]	To assess the feasibility of asynchronous tele-psychiatry and to study the referral patterns	Client education and behaviour change communication	*	*					Service delivery
Chandra et al., 2014 [64]	To assess the acceptability and feasibility of SMS for promoting positive mental health among young women in urban slums	Client education and behaviour change communication	*						Service delivery
Datta et al., 2014 [65]	To assess the feasibility of SMS in delivering MNCH messages in a rural area	Client education and behaviour change communication	*						Service delivery
DeSouza et al., 2014 [66]	To explore the acceptability of delivering healthcare interventions through mobile phones among users in a rural area	Client education and behaviour change communication	*						Service delivery
Gupta et al., 2014 [67]	To evaluate the efficacy of an electronic intensive care unit (ICU) model of service and intervention for the early diagnosis	Provider-to-provider communication	*	*		*			Service delivery

Author	Study Objective	mHealth Tools	Health System Building Blocks#						Primary Health System Building Block
			1	2	3	4	5	6	
	of ST-segment elevation myocardial infarction and prompt initiation of thrombolytic therapy								
Agarwal et al., 2015 [68]	To implement a confidential helpline for increasing access to comprehensive, community based HIV prevention services and improve the HIV/sexually transmitted disease related KAP of men having sex with men	Client education and behaviour change communication	*						Service delivery
Priscilla et al., 2015 [69]	To describe a pragmatic and scalable strategy using mobile technology to promote sustained lifestyle changes to prevent type 2 DM	Client education and behaviour change communication	*	*					Service delivery
Sureshkumar et al., 2015 [70]	To evaluate the feasibility and acceptability of a smartphone enabled, carer supported, educational intervention for management of disability following stroke	Client education and behaviour change communication	*	*					Service delivery
Ajay et al., 2016 [71]	To develop a smartphone enabled hypertension and DM management package to facilitate evidence based care delivery in primary healthcare facilities	Electronic decision support	*	*		*			Service delivery
Sureshkumar et al., 2016 [72]	To evaluate the feasibility and acceptability of a smart phone enabled, carer supported, educational intervention for management of disability following stroke	Client education and behaviour change communication	*	*					Service delivery
Monica et al., 2017 [73]	To assess the feasibility of school hearing screening using telehealth technology	Sensors and point-of-care diagnostics	*			*			Service delivery
Morjaria et al., 2017 [74]	To present the protocol for assessing the effectiveness of a novel mHealth education intervention on spectacle wear among children	Client education and behaviour change communication	*	*					Service delivery
Meher et al., 2009 [75]	To study the awareness and opinions of rural patients towards telemedicine	Client education and behaviour change communication	*	*					Service delivery
Shet et al., 2010 [76]	To explore the pattern of use of mobile phones among patients, and understand their perceptions of a potential mobile phone based intervention to promote adherence to ART	Client education and behaviour change communication	*						Service delivery
Laxmi et al., 2014 [77]	To explore perceptions about using online lifestyle counseling services among individuals living in rural area	Client education and behaviour change communication	*						Service delivery

**Author**	**Study Objective**	**mHealth Tools**	**Health System Building Blocks#**						**Primary Health System Building Block**
			**1**	**2**	**3**	**4**	**5**	**6**	
Priyaa et al., 2014 [78]	To assess perceptions of using SMS as a medium for health information in a rural area	Client education and behaviour change communication	*						Service delivery
Jain et al., 2015 [79]	To gather information related to mobile use patterns, barriers to mobile usage from clients receiving services for severe mental illness	Client education and behaviour change communication	*	*					Service delivery
Ramachandran et al., 2015 [80]	To assess mobile phone usage and willingness to receive health related information among patients attending a chronic disease clinic	Client education and behaviour change communication	*						Service delivery
Rodrigues et al., 2015 [81]	To study perception regarding an mHealth adherence intervention amongst patients on ART	Client education and behaviour change communication	*						Service delivery
Acharya et al., 2016 [82]	To evaluate the patient and doctor perception toward the use of telemedicine	Client education and behaviour change communication	*	*					Service delivery
Mudgapalli et al., 2016 [83]	To explore the perception of receiving health messages through SMS among hypertensive individuals in urban slums	Client education and behaviour change communication	*						Service delivery
Parthaje et al., 2016 [84]	To study the perceptions and knowledge about mHealth among college going students	Client education and behaviour change communication	*						Service delivery
Ramkumar et al., 2016 [85]	To understand parental perceptions and confidence related to tele-audiological diagnostic testing in rural areas	Client education and behaviour change communication	*						Service delivery
Kumari et al., 2006 [86]	To compare patient satisfaction levels between tele-ophthalmology based screening versus conventional diabetic retinopathy screening	Client education and behaviour change communication	*	*					Service delivery
Paul et al., 2006 [87]	To assess patient satisfaction levels and factors influencing it during tele-ophthalmology consultation	Client education and behaviour change communication	*	*					Service delivery
Martinez et al., 2011 [88]	To assess stakeholders' views on exchange of telemedicine services between India and UK	Provider-to-provider communication	*						Service delivery

Author	Study Objective	mHealth Tools	Health System Building Blocks#						Primary Health System Building Block
			1	2	3	4	5	6	
Nahar et al., 2017 [89]	To understand the existing challenges for implementation of mHealth programmes in rural areas	Client education and behaviour change communication	*	*					Service delivery
Deodhar et al., 2002 [90]	To report tele-consultations between a neonatal ICU at an urban hospital and a rural primary care centre	Provider-to-provider communication	*	*					Health workforce
Jialdasani et al., 2006 [91]	To report findings of a tele-pathology network between two rural hospitals and a tertiary care centre	Provider-to-provider communication	*	*					Health workforce
Keeppanasserril et al., 2011 [92]	To find out whether newly graduated dentists, under remote guidance from specialists, can fabricate over-dentures	Provider-to-provider communication	*	*					Health workforce
Mahadevan et al., 2012 [93]	To examine the feasibility, advantages, and disadvantages of tele-education service	Provider training and education		*					Health workforce
Agrawal et al., 2014 [94]	To assess the outcome of telemedicine technology supportive of educational collaboration among endocrine surgery peers and its impact on knowledge and skill development	Provider training and education		*					Health workforce
Thukral et al., 2014 [95]	To evaluate the efficacy of interactive mobile device application 'Apps on sick newborn care' as a training tool, in improving the knowledge and skill scores of postgraduate nursing students	Provider work planning and scheduling	*	*		*			Health workforce
Khanna et al., 2015 [96]	To report the impact of introduction of 'WhatsApp' as an intradepartmental residents communication tool	Provider work planning and scheduling	*	*					Health workforce
Raghu et al., 2015 [97]	To discuss the development and pilot testing of a mobile based, point-of-care clinical decision support tool to assess and manage CVD	Electronic decision support	*	*		*			Health workforce
Chanani et al., 2016 [98]	To measure differences in diagnostic accuracy in community based screening for acute malnutrition using a mobile based solution	Provider work planning and scheduling	*	*					Health workforce
Dorwal et al., 2016 [99]	To assess the role of 'WhatsApp' in the laboratory management system	Provider-to-provider communication	*	*					Health workforce
Kaliyadan et al., 2016 [100]	To evaluate the frequency and types of uses, advantages and disadvantages of 'WhatsApp' based clinical discussion groups	Provider training and education	*	*					Health workforce

Author	Study Objective	mHealth Tools	Health System Building Blocks[#]						Primary Health System Building Block
			1	2	3	4	5	6	
George et al., 2007 [101]	To assess practice, perceptions, awareness and attitudes of doctors towards e-health initiatives	Provider training and education		*					Health workforce
Agarwal et al., 2010 [102]	To gauge the requirements and expectations of doctors from a remote health monitoring system	Provider training and education	*	*					Health workforce
Chattopadhyay et al., 2010 [103]	To survey healthcare staffs' perceptions toward using e-health and to develop a mathematical model to for analysis of overall preparedness of the health centres	Provider training and education	*	*					Health workforce
Gour et al., 2010 [104]	To assess the knowledge, use, and need of computers among healthcare professionals	Provider training and education		*					Health workforce
Boringi et al., 2015 [105]	To assess the knowledge and awareness of tele-dentistry among dental professionals of a dental college	Provider training and education		*					Health workforce
Ganapathy et al., 2016 [106]	To understand the awareness, perception, and attitude of healthcare providers in deploying mHealth	Provider training and education	*	*					Health workforce
Patil et al., 2016 [107]	To explore the attitudes and perceptions of undergraduate students towards mobile phone based learning	Provider training and education		*					Health workforce
Vivek et al., 2016 [108]	To understand the mHealth related KAP of the doctors involved in treatment of acute myocardial infarction in rural areas	Provider-to-provider communication	*	*					Health workforce
Praveen et al., 2014 [109]	To describe the development and field evaluation of a mobile clinical decision support system for CVD in rural India	Electronic decision support	*	*		*			Health workforce
Kaphle et al., 2015 [110]	To develop a framework to assess whether mHealth platforms affect the quality and experience of MNCH care provided by CHWs	Electronic decision support	*	*		*			Health workforce
Modi et al., 2015 [111]	To describe the process of development and formative evaluation of a mHealth intervention to increase the coverage of MNCH services in rural areas by improving the performance of CHWs	Provider work planning and scheduling	*	*					Health workforce
Maulik et al., 2016 [112]	To develop and evaluate the feasibility, acceptability and preliminary effectiveness of a mobile technology based CHW intervention to improve the identification and management of common mental disorders	Electronic decision support	*	*		*			Health workforce

Author	Study Objective	mHealth Tools	Health System Building Blocks#						Primary Health System Building Block
			1	2	3	4	5	6	
Maulik et al., 2017 [113]	To evaluate the feasibility and acceptability of the intervention amongst community members, health workers, and other stakeholders, and document preliminary evidence	Electronic decision support	*	*		*			Health workforce
Perumalsamy et al., 2007 [114]	To evaluate the validity of software for reading digital images and grading diabetic retinopathy	Sensors and point-of-care diagnostics	*			*			Medical products, vaccines and technologies
Srinivasan et al., 2012 [115]	To determine the efficacy of a remotely operated computer based logarithmic visual acuity chart	Sensors and point-of-care diagnostics	*			*			Medical products, vaccines and technologies
Gupta et al., 2013 [116]	To compare the level of agreement, sensitivity and specificity of diagnosis and management decisions of various eye diseases by tele-ophthalmology using indigenous equipment, compared to the in-clinic assessment	Sensors and point-of-care diagnostics	*	*		*			Medical products, vaccines and technologies
Raman et al., 2014 [117]	To compare the accuracy of a tele-screening project with the traditional camp based screenings using single field fundus photography	Sensors and point-of-care diagnostics	*			*			Medical products, vaccines and technologies
Sreelatha et al., 2014 [118]	To assess the efficacy of telephonic visual acuity examination in a hospital based population	Sensors and point-of-care diagnostics	*	*		*			Medical products, vaccines and technologies
Rajalakshmi et al., 2015 [119]	To evaluate the sensitivity and specificity of a smartphone based retinal imaging system, as a screening tool for diabetic retinopathy detection	Sensors and point-of-care diagnostics	*			*			Medical products, vaccines and technologies
Ryan et al., 2015 [120]	To compare smartphone fundus photography, non-mydriatic fundus photography, and 7 field mydriatic fundus photography for their abilities to detect and grade diabetic retinopathy	Sensors and point-of-care diagnostics	*			*			Medical products, vaccines and technologies
Imtiaz et al., 2017 [121]	To investigate the effectiveness, efficiency and cost gains in collecting patient eye health information by trained field investigators through an android based app	Data Collection and Reporting	*	*		*			Medical products, vaccines and technologies
Bedekar et al., 2014 [122]	To determine intra and inter-rater reliability of mobile device goniometer in measuring lumbar flexion range of motion	Sensors and point-of-care diagnostics	*			*			Medical products, vaccines and technologies

**Author**	**Study Objective**	**mHealth Tools**	**Health System Building Blocks#**						**Primary Health System Building Block**
			**1**	**2**	**3**	**4**	**5**	**6**	
Malhotra et al., 2014 [123]	To present the development and preliminary results of diagnostic validation of the tele-psychiatry application, intended for use among adult patients	Sensors and point-of-care diagnostics	*	*		*			Medical products, vaccines and technologies
Singh et al., 2014 [124]	Clinical validation of handheld tele- electrocardiography as a screening tool for evaluation of cardiac diseases in the rural population	Sensors and point-of-care diagnostics	*			*			Medical products, vaccines and technologies
Patterson et al., 2015 [125]	To validate a phone app for epilepsy diagnosis	Electronic decision support	*	*		*			Medical products, vaccines and technologies
Shrivastav et al., 2014 [126]	To understand patient experience with a patch-like external loop recorder for cardiac arrhythmia detection	Sensors and point-of-care diagnostics	*			*			Medical products, vaccines and technologies
Negandhi et al., 2016 [127]	To assess the implementation process of mobile based vaccine management tool	Supply chain management	*	*		*			Medical products, vaccines and technologies
Giduthuri et al., 2014 [129]	To develop and validate a tablet version of an illness explanatory model interview for a public health survey	Data Collection and Reporting			*				Health information system
Krishnan et al., 2010 [128]	To evaluate the effectiveness of a computerized health management information system in rural areas	Electronic Health Records			*				Health information system
Radhakrishna et al., 2014 [130]	To describe the process of design, development, and implementation of a multimodal data portability solution in a rural primary health center	Electronic Health Records			*				Health information system
Diwan et al., 2015 [131]	To describe a mobile based syndromic surveillance system and its application in collecting data on patients' symptoms from formal and informal health care providers	Electronic Health Records			*				Health information system
Pathak et al., 2015 [132]	To assess the feasibility of using mobile phone technology in completing a 30 day follow-up of surgical site infection	Client education and behaviour change communication	*		*				Health information system
Velayutham et al., 2015 [133]	To assess the usefulness and feasibility of mobile interface in tuberculosis notification (MITUN) voice based system for notification of tuberculosis by private medical practitioners	Registries/vital event tracking		*	*				Health information system

**Author**	**Study Objective**	**mHealth Tools**	**Health System Building Blocks**[#]						**Primary Health System Building Block**
			**1**	**2**	**3**	**4**	**5**	**6**	
Kazi et al., 2016 [134]	To evaluate the feasibility of mobile technologies for conducting disease surveillance and monitoring resource utilization at Kumbh Mela	Registries/vital event tracking	*	*	*				Health information system
Modi et al., 2016 [135]	To assess the completeness of pregnancy, delivery, and death registration by CHWs	Registries/vital event tracking	*	*	*			*	Health information system
Negandhi et al., 2016 [136]	To report the process of implementation of tablet based maternal and child tracking	Registries/vital event tracking	*	*	*				Health information system
Gupta et al., 2017 [137]	To test the feasibility of monitoring daily eating pattern among healthy adults with no diagnosed disease using camera phones	Data Collection and Reporting			*				Health information system
Bradley et al., 2012 [138]	To assess the feasibility of using mobile phone technology for sexual behaviour research in a population vulnerable to HIV	Data Collection and Reporting	*		*				Health information system
Taneja et al., 2007 [139]	To identify the factors influencing the effectiveness of an electronic healthcare strategy	Data Collection and Reporting	*	*				*	Leadership and governance
Meher et al., 2013 [140]	To understand the legal issues related to tele-consultations, through doctors' survey at a tertiary care hospital	Provider training and education	*	*				*	Leadership and governance
Jaroslawski et al., 2014 [141]	To understand the kinds of eHealth programmes, the challenges faced and the nature of their financing	Client education and behaviour change communication	*	*			*	*	Leadership and governance
